# The role of spleen radiomics model for predicting prognosis in esophageal squamous cell carcinoma patients receiving definitive radiotherapy

**DOI:** 10.1111/1759-7714.15276

**Published:** 2024-03-13

**Authors:** Longxiang Guo, Ao Liu, Xiaotao Geng, Zongxing Zhao, Yu Nie, Lu Wang, Defeng Liu, Yi Li, Yuanlin Li, Dianxing Li, Qiankun Wang, Zhichao Li, Xiuli Liu, Minghuan Li

**Affiliations:** ^1^ Department of Radiation Oncology Shandong Cancer Hospital, Cheeloo College of Medicine, Shandong University Jinan China; ^2^ Department of Radiation Oncology Shandong Cancer Hospital and Institute, Shandong First Medical University and Shandong Academy of Medical Sciences Jinan China; ^3^ Cheeloo College of Medicine, Shandong University Jinan China; ^4^ Department of Radiation Oncology Qilu Hospital, Cheeloo College of Medicine, Shandong University Jinan China; ^5^ Department of Radiation Oncology Weifang People's Hospital Weifang China; ^6^ Department of Radiation Oncology Liaocheng People's Hospital, Shandong First Medical University Liaocheng China; ^7^ Department of Tumor Radiotherapy Shandong Second Provincial General Hospital Ji'nan China; ^8^ School of Clinical Medicine, Weifang Medical University Weifang China

**Keywords:** esophageal squamous cell cancer, radiomics, spleen, computed tomography, prognosis

## Abstract

**Background:**

The spleen plays an important role in systemic antitumor immune response, but whether spleen imaging features have predictive effect for prognosis and immune status was unknown. The aim of this study was to investigate computed tomography (CT)‐based spleen radiomics to predict the prognosis of patients with esophageal squamous cell carcinoma (ESCC) underwent definitive radiotherapy (dRT) and to try to find its association with systemic immunity.

**Methods:**

This retrospective study included 201 ESCC patients who received dRT. Patients were randomly divided into training (*n* = 142) and validation (*n* = 59) groups. The pre‐ and delta‐radiomic features were extracted from enhanced CT images. LASSO‐Cox regression was used to select the radiomics signatures most associated with progression‐free survival (PFS) and overall survival (OS). Independent prognostic factors were identified by univariate and multivariate Cox analyses. The ROC curve and C‐index were used to evaluate the predictive performance. Finally, the correlation between spleen radiomics and immune‐related hematological parameters was analyzed by spearman correlation analysis.

**Results:**

Independent prognostic factors involved TNM stage, treatment regimen, tumor location, pre‐ or delta‐Rad‐score. The AUC of the delta‐radiomics combined model was better than other models in the training and validation groups in predicting PFS (0.829 and 0.875, respectively) and OS (0.857 and 0.835, respectively). Furthermore, some spleen delta‐radiomic features are significantly correlated with delta‐ALC (absolute lymphocyte count) and delta‐NLR (neutrophil‐to‐lymphocyte ratio).

**Conclusions:**

Spleen radiomics is expected to be a useful noninvasive tool for predicting the prognosis and evaluating systemic immune status for ESCC patients underwent dRT.

## Graphical abstract

To investigate CT‐based spleen radiomics to predict the prognosis of patients with esophageal squamous cell carcinoma (ESCC) underwent definitive radiotherapy (dRT). A total of 201 ESCC patients underwent dRT (training group, *n* = 142, validation group, *n* = 59). The spleen radiomic features extracted from enhanced CT images to calculate spleen Rad‐score, the combination of spleen Rad‐score and clinical factors to establish prognosis prediction model. Independent prognostic factors to predict PFS and OS included TNM stage, treatment regimens, tumor location and spleen Rad‐score. The combined model of spleen Rad‐score and clinical characteristics achieved good effect for predicting PFS and OS. Spleen radiomics is expected to be a useful noninvasive tool for predicting the prognosis for ESCC patients underwent dRT.

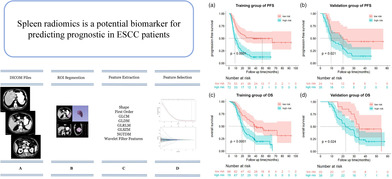

## INTRODUCTION

Esophageal cancer (EC) is one of the most prevalent malignant tumors and the sixth most common cause of cancer‐related deaths worldwide.^1^, ^2^ Currently, definitive chemoradiotherapy (dCRT) is the standard care for unresectable locally advanced EC.^3^ However, more than 50% of patients with ESCC ultimately experience disease progression after dCRT, with a 3‐year progression‐free survival (PFS) rate of only 25%–33%.^4^ The prediction of disease progression and survival helps physicians provide personalized treatment.

However, with tumor being a systemic disease, several studies have revealed the critical impact of systemic immune responses that drive tumor rejection, which may be more important than local antitumor immunity. The spleen is the body's largest secondary lymphoid organ (SLO) and serves as a major filter for blood‐borne pathogens and antigens. It can adjust innate and adaptive immunity, control antigen tolerance, and protect the host or promote disease progression.^5^ As an important immune organ outside the tumor microenvironment, the role of the spleen in tumor–host interactions and tumor progression warrant further investigation. According to related studies, primary tumors can promote tumor progression and metastasis by inducing inflammation and modulating immunosuppression through the spleen,^5^, ^6^ suggesting that the spleen has a close biological relationship with primary tumor development.

In recent years, radiomics has emerged as a novel diagnostic and predictive tool that captures heterogeneity within tissues in a noninvasive manner and converts image data into high‐resolution extractable images using automated, high‐throughput feature‐data extraction algorithms.^7^, ^8^ It is now extensively used to predict the survival prognosis and treatment efficacy of patients with EC.^9^, ^10^, ^11^, ^12^ Previous studies have reported that spleen radiomics predicts prognosis in patients with gastric cancer and recurrence in patients with hepatocellular carcinoma^13^, ^14^; however, none of them further explored its relationship with systemic immunity. We hypothesized that the spleen is a vital part of the immune system and, to some extent, reflects the systemic immunity of the organism. Dynamic variations in splenic structure and function before and after treatment potentially mirror systemic immunity changes, which may be detected using spleen radiomics.

This study aimed to develop a splenic computed tomography (CT)‐based prediction model that integrates radiomic and clinical features for predicting survival in patients with ESCC undergoing definitive radiotherapy (dRT) and dCRT. We also attempted to establish a link between spleen radiomic features and certain peripheral blood immune cell parameters, which, to some extent, reflect the systemic immune status.^15^, ^16^

## METHODS

### Patients

**FIGURE 1:**
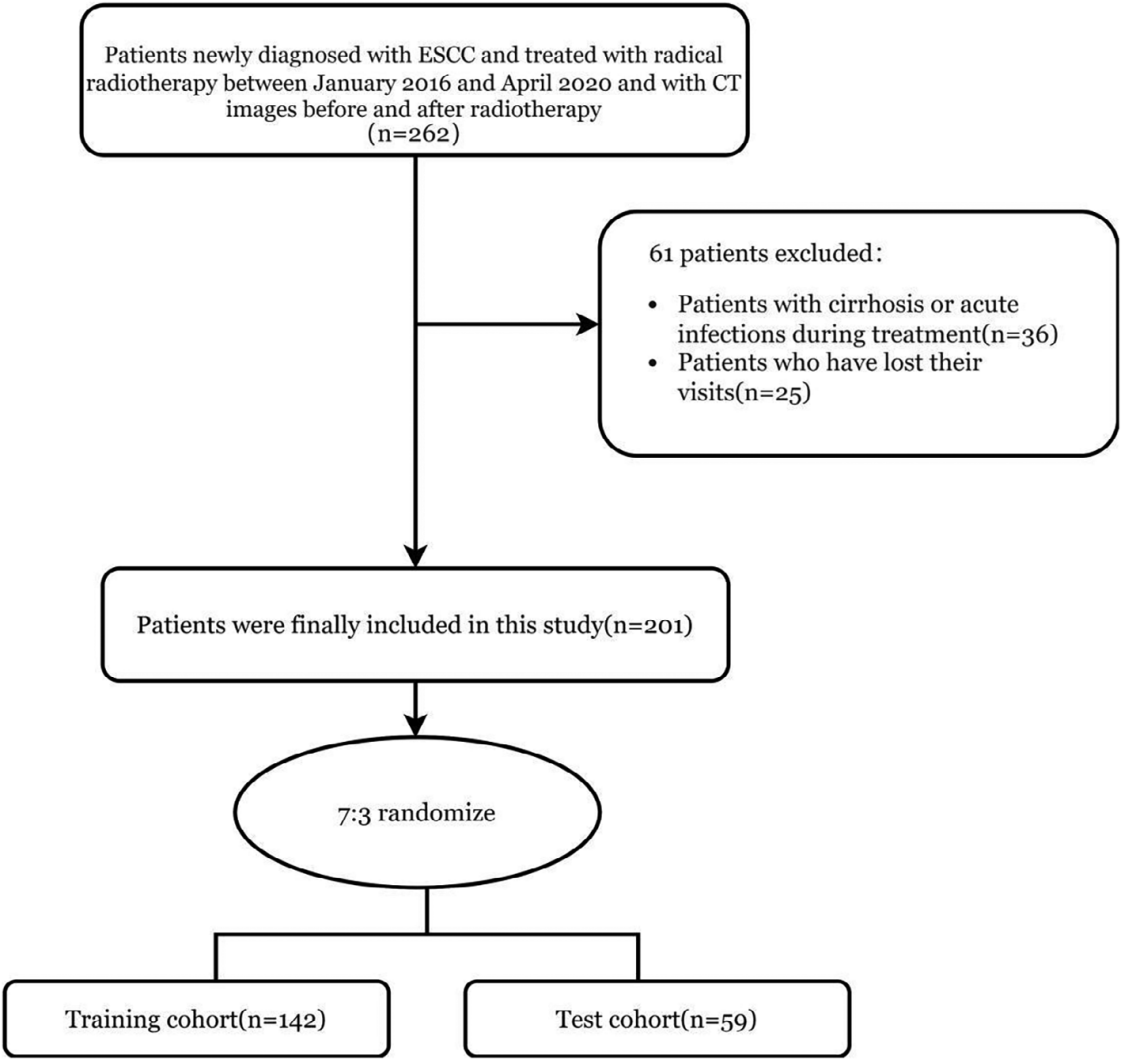
Recruitment and selection process of patients.

**FIGURE 2:**
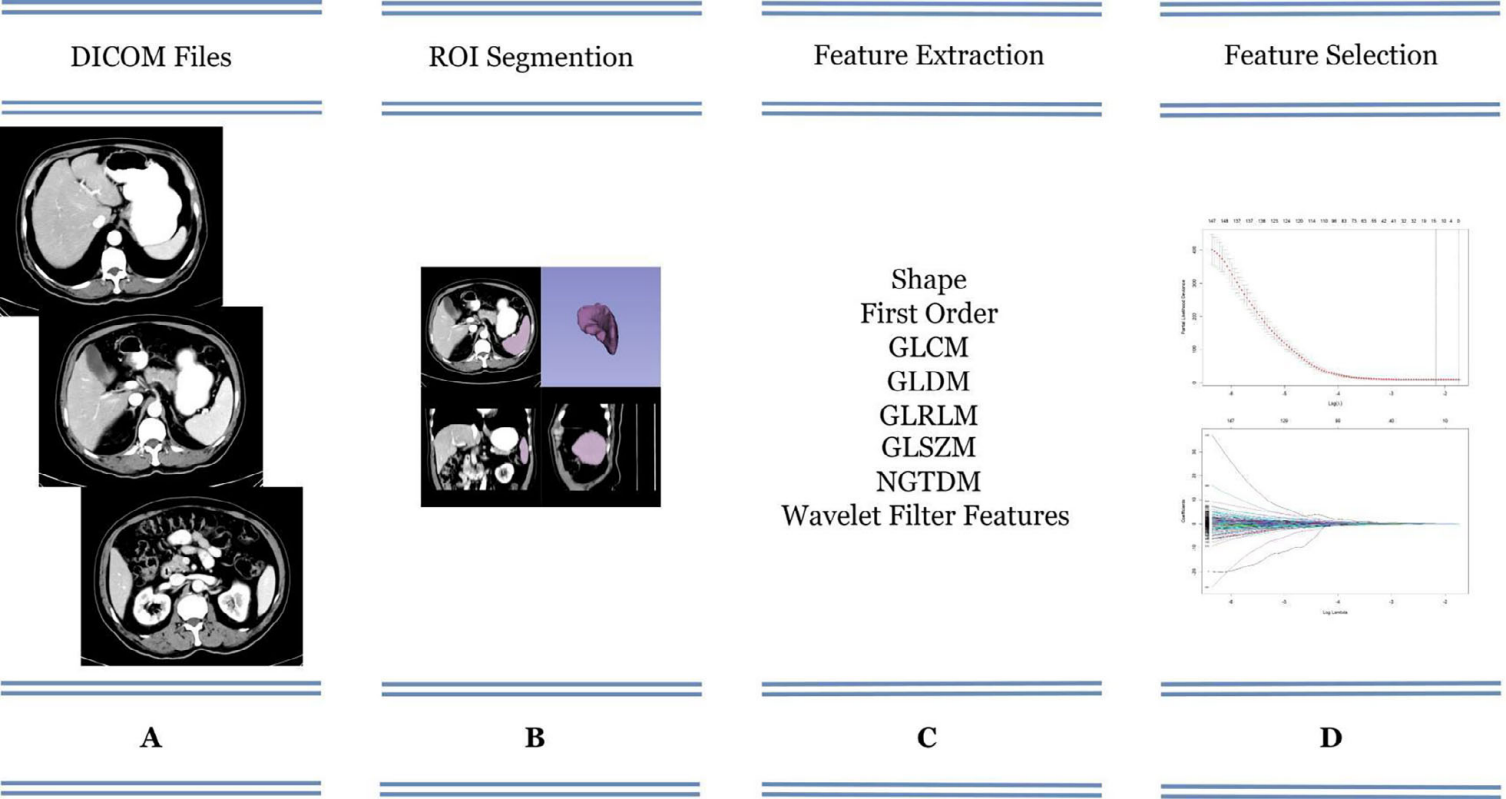
Workflow of the study.

This retrospective study included 201 patients with ESCC treated with definitive radiotherapy (dRT) between January 1, 2016, and April 1, 2020, at Shandong Cancer Hospital and Institute. The inclusion criteria were as follows: (1) Newly diagnosed and pathologically confirmed ESCC; (2) completion of a standard dRT program; (3) age ≤75 years; (4) an Eastern Cooperative Oncology Group performance status score of 0–2; (5) normal liver, kidney, and bone marrow function; and (6) a CT scan of the abdomen 1 month before and 3 months after RT as well as hematology. The exclusion criteria included (1) the presence of other tumors, (2) cirrhosis and splenomegaly, (3) history of previous splenic surgery, (4) the presence of acute infections or autoimmune diseases, (5) long‐term use of hormones, and (6) lost to follow‐up. Finally, all patients who met the criteria were divided into training and validation groups in a 7:3 ratio (Figure 1). The workflow of this study is depicted in Figure 2.

### Ethics statement

Our retrospective study abided by the rules of medical ethics, and the Institutional Review Board (IRB) of Shandong Cancer Hospital approved this study. The number for the ethical statement was SDTHEC201902002. All patients were informed before treatment, agreed to receive concurrent dRT and signed informed consent forms. We protected patient privacy and excluded patient identification information from our analysis.

### Treatment and follow‐up

Most patients with ESCC underwent dCRT. dRT alone was exclusively performed on patients of advanced age, poor physical status, or at their request. All radiation treatments were delivered either as three‐dimensional conformal radiotherapy or intensity modulated radiotherapy with total dose of 50–66 Gy. Radiation was delivered by high‐energy (6 MV) linear accelerators. The gross tumor volume was defined by the primary tumor and any enlarged regional lymph nodes, which were determined using all available information (physical examination, endoscopy, CT). The clinical target volume provided a proximal and distal margin of 3 cm and a 0.5 to 1.0‐cm radical margin and around the gross tumor volume to induce the area of subclinical involvement. The planning target volume was defined as an 8 mm margin of clinical target volume for tumor motion and set‐up variations.^17^, ^18^ Most patients underwent either TP or PF doublet chemotherapy (P refers to a platinum drug such as cisplatin, carboplatin, or oxaliplatin; F refers to a fluoropyrimidine such as 5‐fluorouracil or capecitabine; and T refers to a taxane such as paclitaxel or docetaxel). A few patients received capecitabine or S‐1 monotherapy. In this study, we also assessed the blood parameters of patients including the absolute lymphocyte count (ALC) and neutrophil‐to‐lymphocyte ratio (NLR), before and after treatment, with the time of examination coinciding with the time of CT, and calculated the delta values of the two indicators. The patients were followed up every 3 months in the first 2 years, every 6 months in 3–5 years, and once a year thereafter. Each follow‐up included hematology and standard enhanced CT of the chest and abdomen. PFS was defined as the period from treatment initiation to the initial diagnosis of progression, death, or the final follow‐up. Overall survival (OS) was defined as the period from treatment initiation to the date of death from any cause.

### Imaging acquisition and tumor segmentation

All patients underwent standard chest and abdominal enhanced CT 1 month before treatment and 3 months after treatment. Breath‐holding was done during CT scan to maximize reduce the range of spleen motion. A 64‐layer spiral CT scanner (Definition AS+; Siemens SOMATOM) was used for CT image acquisition. The scanning parameters were as follows: a slice thickness of 5.0 mm, tube voltage of 120 kV, and tube current of 220 mA. We selected to outline the spleen from the venous phase images. Enhanced CT images in DICOM format were extracted from the PACS (picture archiving and communication system) and used for feature extraction.

The CT images were uploaded to 3D Slicer software (version 5.0.3, USA) to semi‐automatically outline the splenic region, intentionally avoiding the large splenic vessels during outlining. The segmentation was then manually modified on each slice of the CT image by a radiologist, and the final segmentation results were examined by another radiologist.

### Radiomic features extraction/selection and Rad‐score construction

Patients were randomly assigned to the training or validation groups. We ensured that the patient ratio in the training and validation groups was 7:3. We obtained 1037 features on each of the pre‐ and post‐treatment CT images using PyRadiomics, an open‐source package in 3D‐Slicer software, including seven types of features: shape, first‐order, gray level cooccurrence matrix, gray level run length matrix, gray level size zone matrix, gray level dependence matrix, and neighborhood gray‐tone difference matrix (https://pyradiomics.readthedocs.io/). The delta‐radiomic features were obtained by subtracting the pre‐ from the post‐treatment features.

Thirty patients were then randomly selected from the training group, and their spleens were resketched by another experienced radiologist for use in intragroup correlation coefficient (ICC) analysis. Finally, features with ICC values ≥0.75 were those with favorable reproducibility, and they were used in the subsequent analysis step. Data standardization was achieved using the Z‐score, a means of uniformly converting data of different magnitudes to the same magnitude to ensure comparability between data. Least absolute shrinkage and selection operator (LASSO) Cox regression was subsequently used to select the features from the training group that were most relevant to PFS and OS in patients with ESCC.^19^ Parameter tuning was performed under 10‐fold cross‐validation to obtain the optimal number of features and avoid overfitting. Finally, the radiomic signatures were determined based on the performance of the LASSO Cox regression. Each patient's radiomics score (Rad‐score) was a linear combination of the radiomic features and associated weights.^20^, ^21^

### Construction and validation of survival prediction models

We constructed five models, including a clinical model; delta‐radiomics model; pre‐radiomics model; and two models with varying combinations of clinical, delta‐radiomics, and preradiomic features, to predict PFS and OS separately via multivariate Cox regression. Finally, the best‐performing model was selected to construct a nomogram for predicting survival, thus achieving a more intuitive prediction of survival. Consequently, according to the Rad‐score, patients were divided into high‐ and low‐risk groups. After the survival curves were evaluated using the Kaplan–Meier (KM) method, comparisons between the two groups were made using the log‐rank test (*p* < 0.05). The consistency index (C‐index) based on receiver operating characteristic (ROC) curves was used to assess the performance of the five models. The C‐index ranges from 0.5 to 1.0, with larger values representing superior predictive performance.^22^ Calibration curves were quoted to evaluate agreement between nomogram predictions and actual patient outcomes.^23^

### Statistical analysis

The baseline characteristics of patients were categorized into continuous (hematological parameters) and categorical variables. Differences between the training and validation groups were evaluated using the *t*‐test (normally distributed continuous variables), Mann–Whitney U test (non‐normally distributed continuous variables), and chi‐square test (categorical variables). The parameters used for data analysis and model construction were complete and did not require resolution of missing values. All data were analyzed using SPSS (version 26.0, IBM) and R (version 4.2.2, http://www.project.org/) software. All statistical tests were two‐sided, and statistical significance was set at *p* < 0.05.

## RESULTS

### Patient characteristics

A total of 201 patients were enrolled and randomly divided into the training (*n* = 142) and validation (*n* = 59) groups (Table 1). The last time for follow‐up was May 1, 2023. The median PFS and OS were 13.3 (95% CI: 9.652–16.948) and 26.3 (95% CI: 21.102–31.498) months, respectively.

**TABLE 1:** Comparison of patient characteristics between training and validation groups.

Characteristics	Training (*n* = 142)	Validation (*n* = 59)	χ^2^	*p*‐value
Gender, *n* (%)			0.000	0.989
Male	29 (20.4%)	12 (21.3%)		
Female	113 (79.6%)	47 (78.7%)		
Age, *n* (%)			0.243	0.622
<65	81 (57.0%)	31 (52.4%)		
≥65	61 (43%)	28 (47.5%)		
Location, *n* (%)			3.796	0.150
Upper	73 (51.4%)	26 (45.0%)		
Middle	48 (33.8%)	28 (46.7%)		
Down	21 (14.8%)	5 (8.3%)		
Smoking, *n* (%)			0.114	0.736
Yes	83 (58.5%)	36 (37.7%)		
No	59 (41.5%)	23 (62.3%)		
Drinking, *n* (%)			0.300	0.584
Yes	72 (50.7%)	32 (54.1%)		
No	70 (49.3%)	27 (45.9%)		
T stage, *n* (%)			2.065	0.356
2	17 (12%)	6 (9.8%)		
3	89 (62.6%)	43 (72.2%)		
4	36 (25.4)	10 (18.0%)		
N stage, *n* (%)			0.227	0.973
0	28 (19.7%)	10 (19.7%)		
1	71 (50.0%)	30 (49.2%)		
2	36 (25.4%)	16 (26.2%)		
3	7 (4.9%)	3 (4.9%)		
TNM stage, *n* (%)			1.234	0.540
II	29 (20.4%)	12 (21.3%)		
III	74 (52.1%)	35 (57.4%)		
IV	39 (27.5%)	12 (21.3%)		
Treatment regimen, *n* (%)			0.445	0.505
CRT	120 (84.5%)	52 (86.9%)		
RT	22 (15.5%)	7 (13.1%)		
Chemotherapy, *n* (%)			4.486	0.214
TP	60 (42.3%)	34 (3.3%)		
PF	48 (33.8%)	16 (26.2%)		
Other	12 (8.5%)	2 (57.4%)		
Radiation dose, *n* (%)			2.073	0.150
<59.4 Gy	43 (30.3%)	12 (21.3%)		
≥59.4 Gy	99 (69.7%)	47 (78.7%)		
ALC, median (×10^3^ cells/μL)				
Pre	1.74 (0.65–2.96)	1.76 (0.72–2.99)		0.132
Post	0.81 (0.23–2.26)	0.91 (0.1–2.92)		0.503
Nadir	0.33 (0.03–1.5)	0.32 (0.1–1.27)		0.528
Delta	−0.86 (−2.28–0.58)	−0.80 (−2.79–0.59)		0.685
NLR, median (×10^3^)				
Pre	2.11 (0.84–9.78)	2.12 (0.22–8.74)		0.521
Post	3.34 (0.75–43.86)	2.93 (0.88–31.3)		0.551
Delta	0.97 (−8.85–41.82)	0.58 (−5.53–27.58)		0.761

Abbreviations: ALC, absolute lymphocyte count; CRT, chemoradiotherapy; Gy, gray; NLR, neutrophil‐to‐lymphocyte ratio; RT, radiotherapy; TNM, tumor–node–metastasis.

### Clinicopathological prognostic factors

Univariate analyses were performed for all clinical parameters in the training group. Only variables with *p* < 0.1 in the univariate regression analysis were included in the multivariate analysis. After multivariate analysis, statistically significant factors included tumor–node–metastasis (TNM) stage and treatment regimen for predicting PFS and TNM stage, treatment regimen, tumor location, and pre‐NLR for predicting OS (Table 2).

**TABLE 2:** PFS and OS‐related univariate and multivariate analysis in the training group.

	PFS				OS			
Variables	Training cohort				Training cohort			
	Univariate analysis		Multivariate analysis		Univariate analysis		Multivariate analysis	
	HR (95% CI)	*p*‐value	HR (95% CI)	*p*‐value	HR (95% CI)	*p*‐value	HR (95% CI)	*p*‐value
Clinical characteristic								
Sex	1.439 (0.853–2.428)	0.173			0.686 (0.406–1.157)	0.157		
Age	0.931 (0.624–1.390)	0.727			1.050 (0.703–1.568)	0.812		
Smoking	0.847 (0.566–1.266)	0.418			1.267 (0.846–1.897)	0.252		
Drinking	1.425 (0.958–2.118)	0.080	1.287 (0.837–1.979)	0.251	1.450 (0.973–2.162)	0.068	1.344 (0.870–2.075)	0.183
Tumor location								
Upper	Reference				Reference		Reference	
Middle	1.416 (0.502–0.888)	0.116			1.433 (0.928–2.213)	0.104	1.366 (0.837–2.228)	0.212
Down	1.581 (0.502–0.888)	0.115			2.031 (1.143–3.609)	0.016	2.093 (1.101–3.979)	0.024
T stage								
2	Reference		Reference		Reference		Reference	
3	3.668 (1.473–9.132)	0.005	1.412 (0.440–4.525)	0.562	3.571 (1.430–8.919)	0.006	0.921 (0.267–3.177)	0.896
4	6.679 (2.574–17.330)	<0.0001	0.405 (0.065–2.516)	0.332	8.115 (3.103–21.222)	<0.0001	0.380 (0.054–2.679)	0.331
N stage								
0	Reference		Reference		Reference		Reference	
1	4.510 (2.220–9.160)	<0.0001	1.721 (0.716–4.135)	0.225	4.405 (2.092–9.275)	<0.0001	1.353 (0.543–3.370)	0.516
2	4.249 (1.999–9.033)	<0.0001	1.369 (0.544–3.449)	0.505	4.991 (2.267–10.986)	<0.0001	1.190 (0.455–3.111)	0.722
3	6.761 (2.390–19.121)	<0.0001	0.781 (0.188–3.233)	0.733	7.621 (2.612–22.231)	<0.0001	0.432 (0.097–1.925)	0.271
TNM stage								
II	Reference		Reference		Reference		Reference	
III	9.535 (3.809–23.869)	<0.0001	5.220 (1.456–18.714)	0.011	11.320 (4.088–31.343)	<0.0001	9.381 (2.253–39.063)	0.002
IV	14.005 (5.413–36.237)	<0.0001	32.258 (4.2797–243.158)	0.001	20.643 (7.202–59.167)	<0.0001	44.957 (5.021–402.573)	0.001
Treatment regimen	2.259 (1.361–3.747)	0.002	2.201 (1.252–3.871)	0.006	2.424 (1.456–4.033)	0.001	2.225 (1.232–4.019)	0.008
RT dose	0.623 (0.410–0.945)	0.026	0.660 (0.421–1.035)	0.070	0.644 (0.424–0.978)	0.039	0.705 (0.445–1.118)	0.138
Pre‐ALC	0.672 (0.443–1.020)	0.062	0.754 (0.460–1.236)	0.263	0.700 (0.455–1.076)	0.104		
Post‐ALC	0.762 (0.472–1.229)	0.265			0.746 (0.460–1.209)	0.234		
ALC nadir	1.046 (0.418–2.618)	0.923			1.112 (0.442–2.796)	0.821		
Delta‐ALC	1.169 (0.797–1.716)	0.425			1.105 (0.743–1.645)	0.621		
Pre‐NLR	1.136 (1.005–1.285)	0.042	1.650 (0.928–1.223)	0.370	1.152 (1.018–1.303)	0.024	1.147 (1.007–1.308)	0.040
Post‐NLR	1.031 (0.999–1.063)	0.055	1.009 (0.976–1.044)	0.579	1.029 (0.998–1.062)	0.069	1.489 (0.966–2.296)	0.072
Delta‐NLR	1.022 (0.989–1.056)	0.195			1.020 (0.987–1.055)	0.238		
Clinical characteristic and delta‐Rad‐score								
Sex	1.439 (0.853–2.428)	0.173			0.686 (0.406–1.157)	0.157		
Age	0.931 (0.624–1.390)	0.727			1.050 (0.703–1.568)	0.812		
Smoking	0.847 (0.566–1.266)	0.418			1.267 (0.846–1.897)	0.252		
Drinking	1.425 (0.958–2.118)	0.080	1.445 (0.928–2.250)	0.103	1.450 (0.973–2.162)	0.068	1.539 (0.980–2.415)	0.061
Tumor location								
Upper	Reference				Reference		Reference	
Middle	1.416 (0.502–0.888)	0.116			1.433 (0.928–2.213)	0.104	1.410 (0.858–2.317)	0.175
Down	1.581 (0.502–0.888)	0.115			2.031 (1.143–3.609)	0.016	2.437 (1.285–4.623)	0.006
T stage								
2	Reference		Reference		Reference		Reference	
3	3.668 (1.473–9.132)	0.005	1.354 (0.433–4.236)	0.603	3.571 (1.430–8.919)	0.006	1.050 (0.318–3.468)	0.936
4	6.679 (2.574–17.330)	<0.0001	0.619 (0.100–3.834)	0.606	8.115 (3.103–21.222)	<0.0001	0.430 (0.060–3.084)	0.401
N stage								
0	Reference		Reference		Reference		Reference	
1	4.510 (2.220–9.160)	<0.0001	1.667 (0.692–4.018)	0.255	4.405 (2.092–9.275)	<0.0001	1.576 (0.623–3.987)	0.336
2	4.249 (1.999–9.033)	<0.0001	1.428 (0.563–3.622)	0.453	4.991 (2.267–10.986)	<0.0001	1.510 (0.559–4.082)	0.417
3	6.761 (2.390–19.121)	<0.0001	1.198 (0.286–5.023)	0.805	7.621 (2.612–22.231)	<0.0001	0.656 (0.142–3.020)	0.588
TNM stage								
II	Reference		Reference		Reference		Reference	
III	9.535 (3.809–23.869)	<0.0001	4.065 (1.123–14.717)	0.033	11.320 (4.088–31.343)	<0.0001	6.893 (1.667–28.506)	0.008
IV	14.005 (5.413–36.237)	<0.0001	12.913 (1.656–100.715)	0.015	26.498 2.922–240.283)	<0.0001	16.687 (1.717–162.217)	0.004
Treatment regimen	2.259 (1.361–3.747)	0.002	2.087 (1.192–3.654)	0.010	2.424 (1.456–4.033)	0.001	1.867 (1.010–3.449)	0.046
RT dose	0.623 (0.410–0.945)	0.026	0.634 (0.401–1.003)	0.052	0.644 (0.424–0.978)	0.039	0.710 (0.443–1.140)	0.156
Pre‐ALC	0.672 (0.443–1.020)	0.062	0.969 (0.584–1.609)	0.904	0.700 (0.455–1.076)	0.104		
Post‐ALC	0.762 (0.472–1.229)	0.265			0.746 (0.460–1.209)	0.234		
ALC nadir	1.046 (0.418–2.618)	0.923			1.112 (0.442–2.796)	0.821		
Delta‐ALC	1.169 (0.797–1.716)	0.425			1.105 (0.743–1.645)	0.621		
Pre‐NLR	1.136 (1.005–1.285)	0.042	1.020 (0.889–1.172)	0.775	1.152 (1.018–1.303)	0.024	1.068 (0.927–1.229)	0.362
Post‐NLR	1.031 (0.999–1.063)	0.055	1.011 (0.976–1.048)	0.538	1.029 (0.998–1.062)	0.069	0.992 (0.955–1.030)	0.665
Delta‐NLR	1.022 (0.989–1.056)	0.195			1.020 (0.987–1.055)	0.238		
Delta‐Rad‐score	57.393 (15.547–211.870)	<0.0001	15.195 (3.153–73.220)	0.001	27.721 (7.903–97.234)	<0.0001	11.747 (2.789–49.484)	0.001
Clinical characteristic and pre‐Rad‐score								
Sex	1.439 (0.853–2.428)	0.173			0.686 (0.406–1.157)	0.157		
Age	0.931 (0.624–1.390)	0.727			1.050 (0.703–1.568)	0.812		
Smoking	0.847 (0.566–1.266)	0.418			1.267 (0.846–1.897)	0.252		
Drinking	1.425 (0.958–2.118)	0.080	1.320 (0.858–2.031)	0.207	1.450 (0.973–2.162)	0.068	1.327 (0.858–2.052)	0.203
Tumor location								
Upper	Reference				Reference		Reference	
Middle	1.416 (0.502–0.888)	0.116			1.433 (0.928–2.213)	0.104	1.215 (0.726–2.036)	0.459
Down	1.581 (0.502–0.888)	0.115			2.031 (1.143–3.609)	0.016	1.938 (1.023–3.671)	0.042
T stage								
2	Reference	0.005	Reference	0.496	Reference	0.006	Reference	0.952
3	3.668 (1.473–9.132)	<0.0001	1.475 (0.481–4.522)	0.855	3.571 (1.430–8.919)	<0.0001	0.963 (0.282–3.295)	0.923
4	6.679 (2.574–17.330)		0.839 (0.127–5.557)		8.115 (3.103–21.222)		0.898 (0.100–8.036)	
N stage								
0	Reference		Reference		Reference		Reference	
1	4.510 (2.220–9.160)	<0.0001	1.352 (0.554–3.301)	0.508	4.405 (2.092–9.275)	<0.0001	1.185 (0.474–2.958)	0.717
2	4.249 (1.999–9.033)	<0.0001	1.634 (0.640–4.176)	0.305	4.991 (2.267–10.986)	<0.0001	1.405 (0.530–3.724)	0.494
3	6.761 (2.390–19.121)	<0.0001	1.059 (0.245–4.570)	0.939	7.621 (2.612–22.231)	<0.0001	0.477 (0.106–2.151)	0.336
TNM stage								
II	Reference	<0.0001	Reference	0.010	Reference	<0.0001	Reference	0.002
III	9.535 (3.809–23.869)		5.311 (1.493–18.892)		11.320 (4.088–31.343)		9.714 (2.299–41.033)	
IV	14.005 (5.413–36.237)	<0.0001	13.212 (1.639–106.509)	0.015	26.498 2.922–240.283)	<0.0001	18.508 (1.730–197.963)	0.016
Treatment regimen	2.259 (1.361–3.747)	0.002	1.866 (1.056–3.298)	0.032	2.424 (1.456–4.033)	0.001	1.780 (0.970–3.267)	0.063
RT dose	0.623 (0.410–0.945)	0.026	0.718 (0.451–1.144)	0.163	0.644 (0.424–0.978)	0.039	0.803 (0.497–1.298)	0.371
Pre‐ALC	0.672 (0.443–1.020)	0.062	0.733 (0.455–1.182)	0.202	0.700 (0.455–1.076)	0.104		
Post‐ALC	0.762 (0.472–1.229)	0.265			0.746 (0.460–1.209)	0.234		
ALC nadir	1.046 (0.418–2.618)	0.923			1.112 (0.442–2.796)	0.821		
Delta‐ALC	1.169 (0.797–1.716)	0.425			1.105 (0.743–1.645)	0.621		
Pre‐NLR	1.136 (1.005–1.285)	0.042	1.044 (0.915–1.192)	0.520	1.152 (1.018–1.303)	0.024	1.135 (0.994–1.295)	0.061
Post‐NLR	1.031 (0.999–1.063)	0.055	1.016 (0.983–1.05)	0.348	1.029 (0.998–1.062)	0.069	1.003 (0.967–1.040)	0.878
Delta‐NLR	1.022 (0.989–1.056)	0.195			1.020 (0.987–1.055)	0.238		
Pre‐Rad‐score	27.128 (8.526–86.320)	<0.0001	24.351 (6.788–87.356)	<0.0001	47.236 (11.745–189.977)	<0.0001	11.747 (2.789–49.484)	<0.0001

Abbreviations: ALC, absolute lymphocyte counts; CI, confidence interval; HR, hazard ratio; NLR, neutrophil‐to‐lymphocyte ratio; OS, overall survival; PFS, progression‐free survival; RT, radiotherapy; TNM, tumor–node–metastasis.

### Radiomic feature selection

**FIGURE 3:**
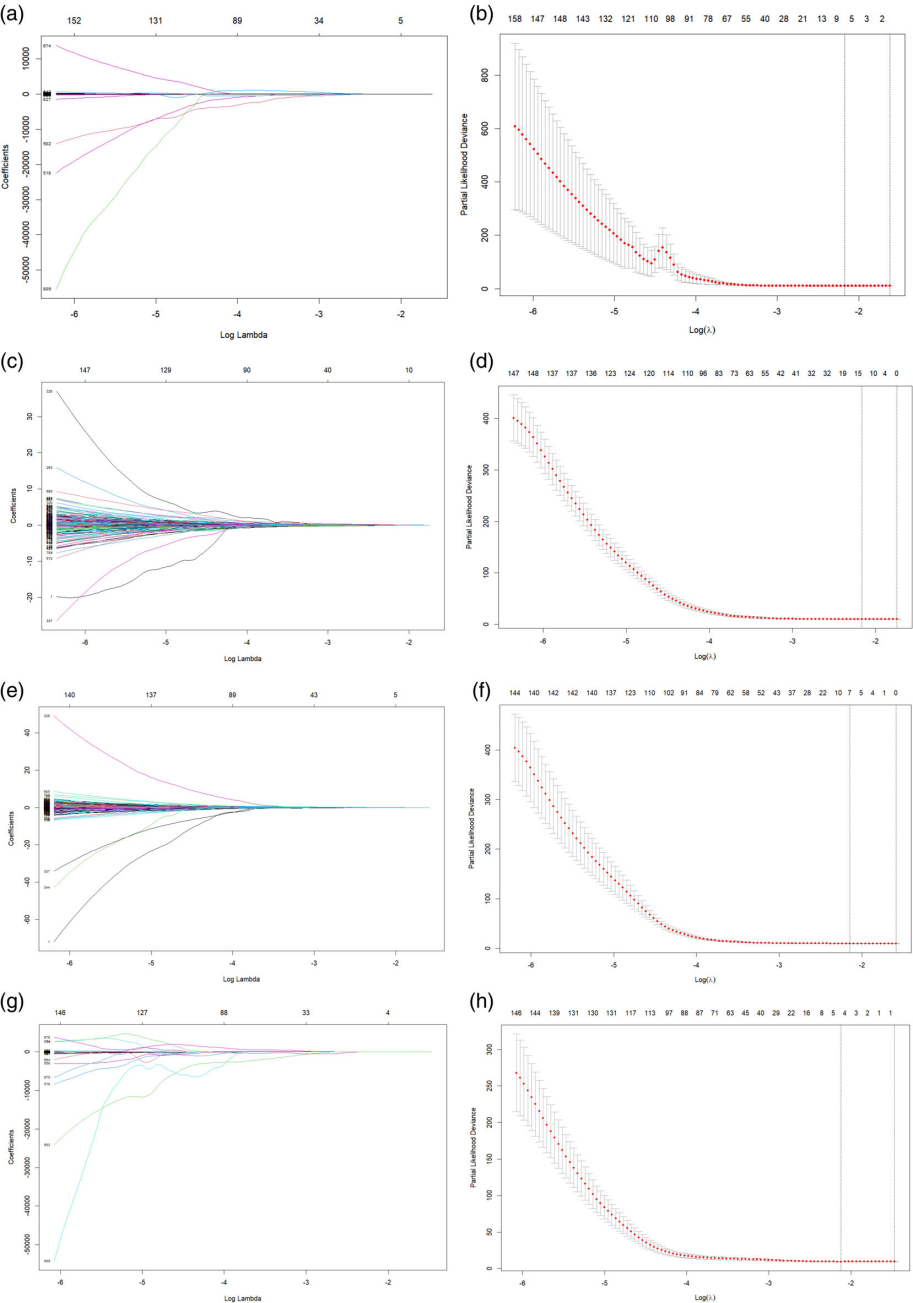
Selection of radiomic features associated with progression‐free survival (PFS) and overall survival (OS) based on LASSO Cox regression models. (a, b) PFS and (e, f) OS are coefficient and crossvalidation curves of the preradiomic features. (c, d) PFS and (g, h) OS are coefficient and crossvalidation curves of the delta‐radiomic features.

A total of 1037 features were extracted. Radiomic features with ICC values ≥0.75 were selected for the subsequent analysis, and they were normalized by the Z‐score. A total of 935 and 823 pre‐ and delta‐radiomic features were retained, respectively. Finally, the radiomic features most pertinent to the patients' PFS and OS were selected by the LASSO algorithm (Figure 3). Among these, the features most relevant to PFS and OS were selected, and their corresponding coefficients determined (Table 3). The radiomic features values were multiplied by their coefficients to obtain the Rad‐score.

**TABLE 3:** Radiomic features associated with PFS and OS selected by LASSO regression.

Radiomic features	Coefficients
Progression‐free survival	
Delta‐radiomic features	
original_shape_MajorAxisLength	−0.076217324
original_shape_Maximum2DDiameterSlice	−0.013740404
original_glrlm_GrayLevelNonUniformityNormalized	0.023101401
log.sigma.3.mm.3D_gldm_LowGrayLevelEmphasis	0.021223857
log.sigma.3.mm.3D_glrlm_GrayLevelNonUniformity	−0.022023095
log.sigma.5.mm.3D_firstorder_Kurtosis	−0.004543919
log.sigma.5.mm.3D_firstorder_Range	−0.006308708
wavelet.LLH_glcm_DifferenceVariance	0.006770692
wavelet.LLH_glrlm_ShortRunLowGrayLevelEmphasis	0.016211680
wavelet.LHL_glszm_LargeAreaEmphasis	−0.028971369
wavelet.HLL_firstorder_Kurtosis	0.102648898
wavelet.HHH_firstorder_Kurtosis	0.086421012
wavelet.HHH_firstorder_Median	−0.042008561
wavelet.HHH_gldm_HighGrayLevelEmphasis	−0.002273581
Preradiomic features	
log.sigma.5.mm.3D_firstorder_Maximum	0.43460715
wavelet.HLL_firstorder_Kurtosis	−0.07659121
wavelet.HHL_firstorder_Maximum	−0.47441403
wavelet.HHL_glcm_Correlation	−0.10983043
wavelet.HHH_gldm_HighGrayLevelEmphasis	22.21596529
Overall survival	
Delta‐radiomic features	
original_shape_Maximum2DDiameterSlice	−0.06910814
original_gldm_GrayLevelNonUniformity	−0.01688211
log.sigma.3.mm.3D_glrlm_GrayLevelNonUniformity	−0.07308556
log.sigma.5.mm.3D_firstorder_Range	−0.00433705
wavelet.LHL_glszm_LargeAreaEmphasis	−0.04377409
wavelet.HLL_firstorder_Kurtosis	0.10280884
wavelet.HHH_firstorder_Kurtosis	0.12678523
Preradiomic features	
log.sigma.5.mm.3D_firstorder_Maximum	0.35326278
wavelet.HLL_ngtdm_Contrast	0.08488541
wavelet.HHL_firstorder_Maximum	−0.50657370
wavelet.HHL_glcm_Correlation	−0.08109222
wavelet.HHH_gldm_GrayLevelNonUniformity	2.84527886

Abbreviations: OS, overall survival; PFS, progresion‐free survival.

### Development and validation of a predictive nomogram based on the Rad‐score

**FIGURE 4:**
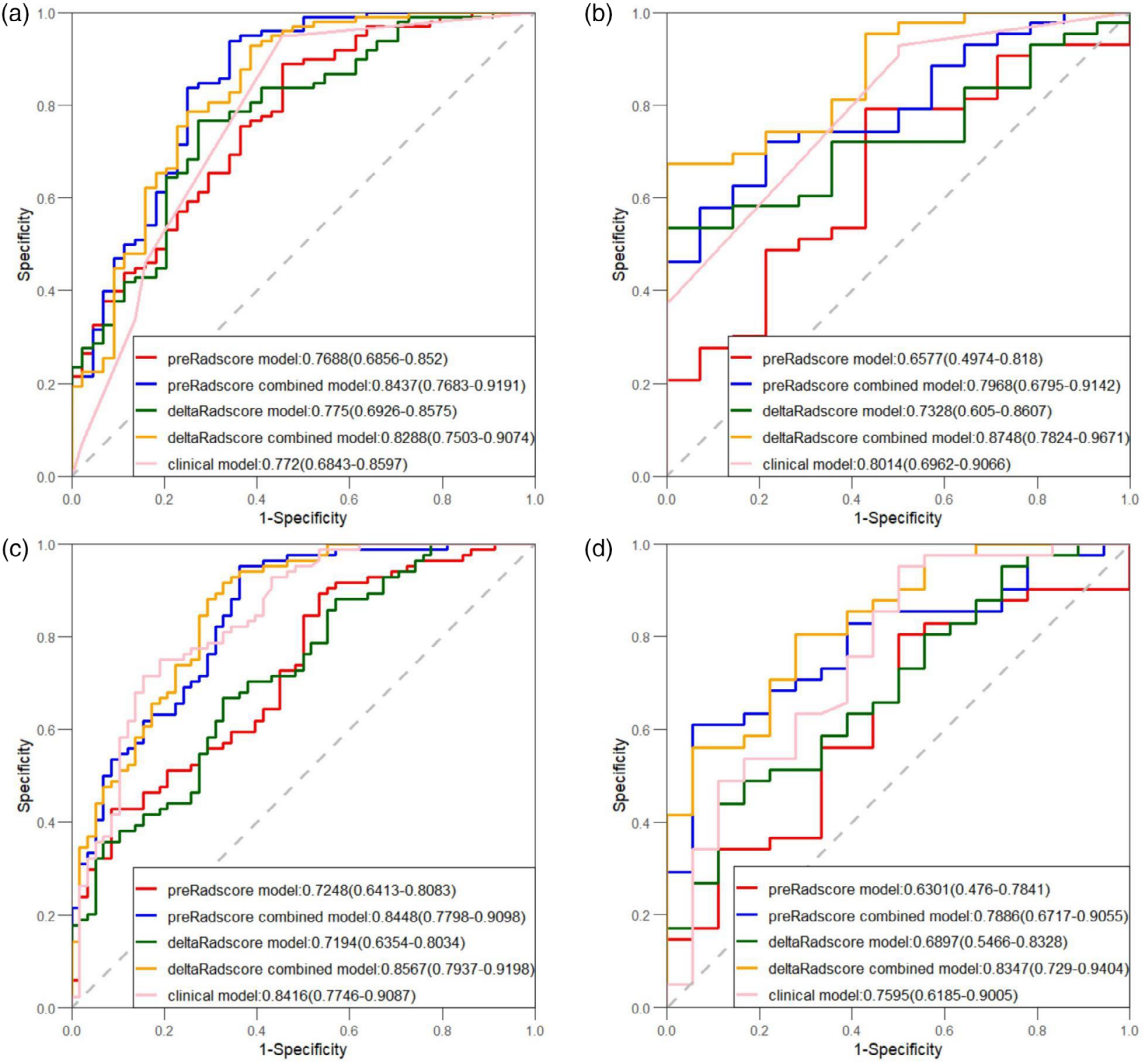
Receiver operating characteristic (ROC) curves constructed based on the five models. (a and b) The ROC curves for predicting progression‐free survival (PFS (in the training and validation groups, respectively. (c and d) The ROC curves for predicting overall survival (OS) in the training and validation groups, respectively.

**FIGURE 5:**
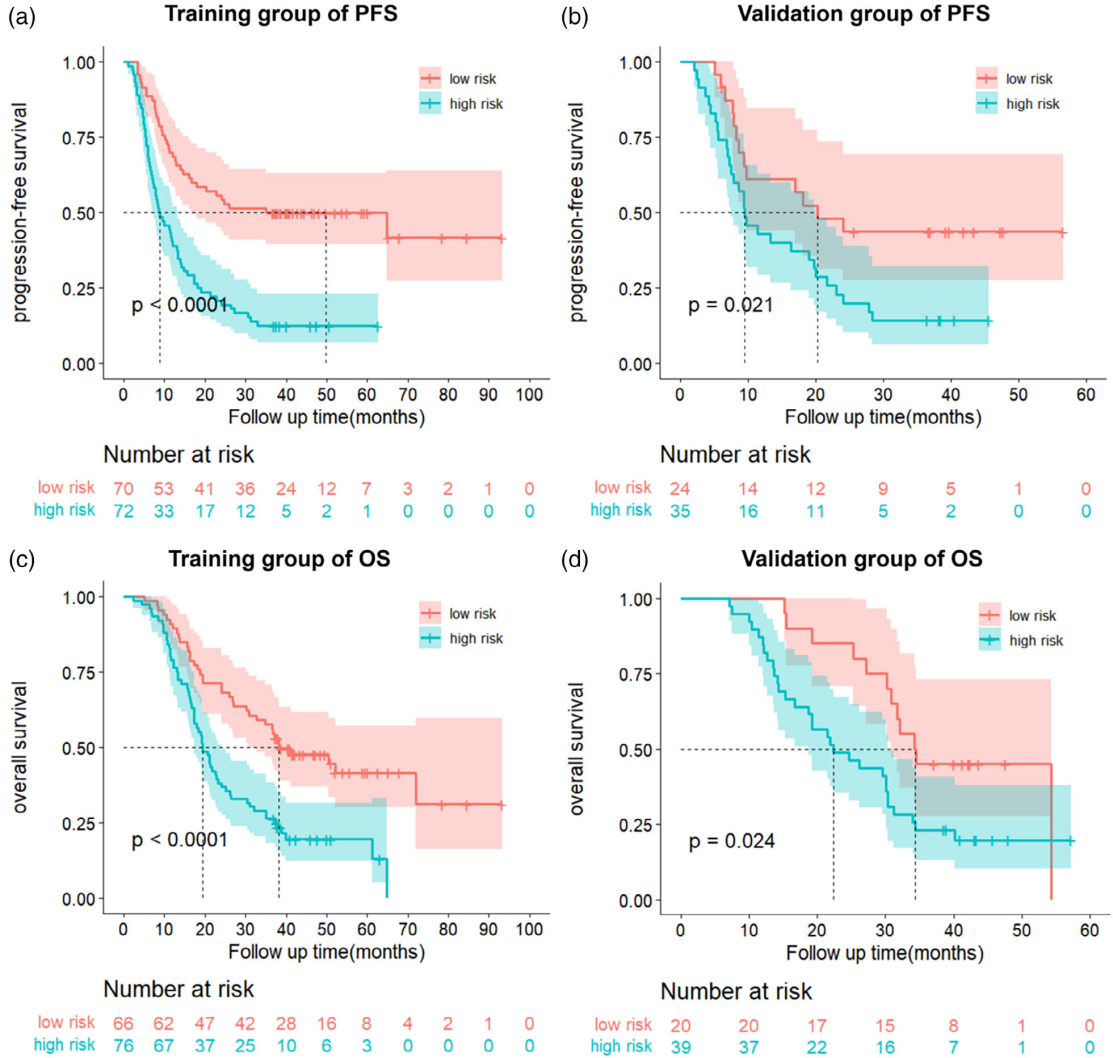
The Kaplan‐Meier (KM) curve of the prediction model. (a) The KM curve in the training group of progression‐free survival (PFS). (b) The KM curve in the validation group of progression‐free survival (PFS). (c) KM curve in training group of overall survival. (d) KM curve in validation group.

**FIGURE 6:**
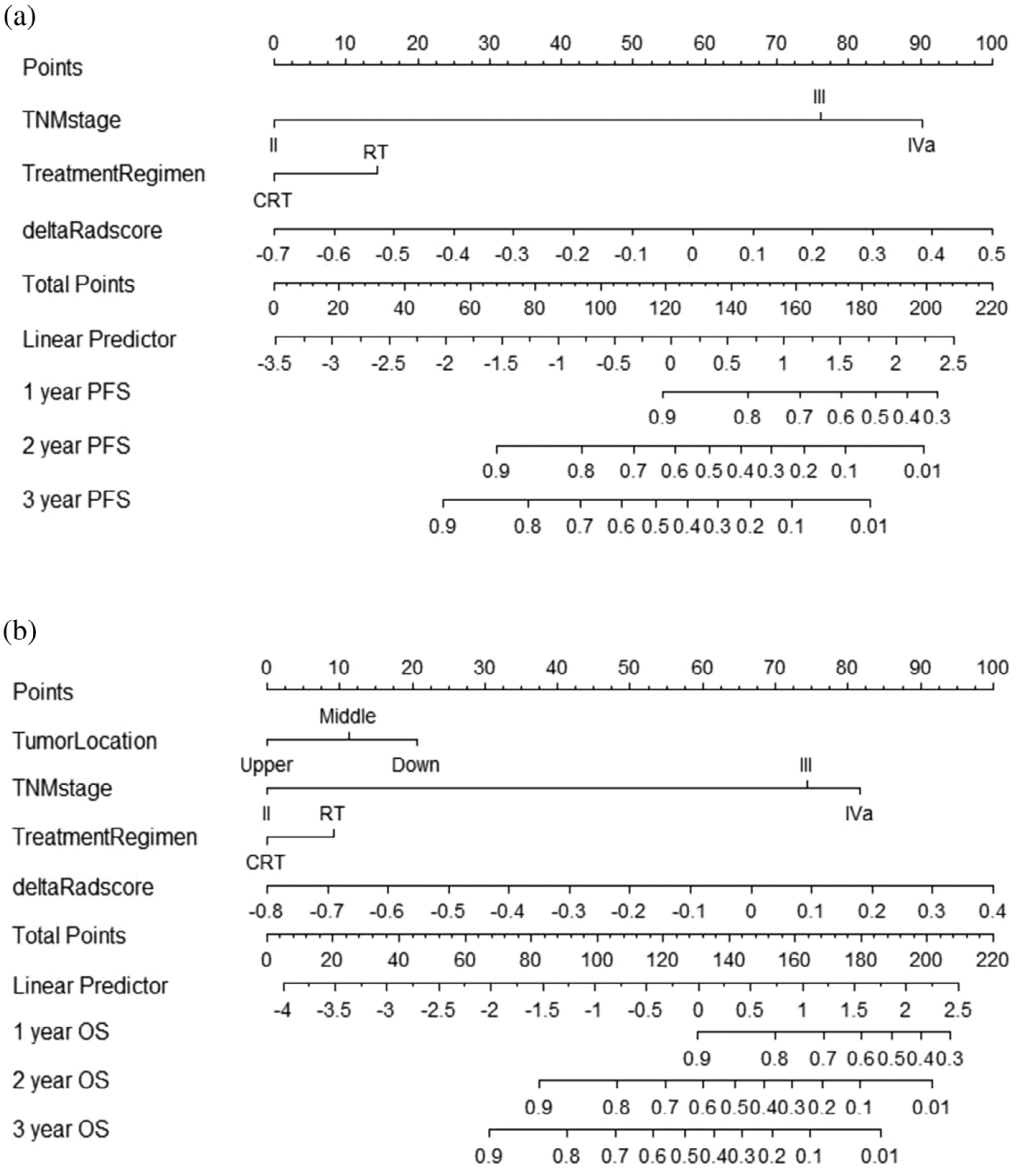
The hybrid radiomics nomogram for predicting progression‐free survival (PFS) (a) and overall survival (OS) (b) based on clinical factors and the splenic delta‐radiomic features. The nomogram allows the user to obtain the probability of 1‐, 2‐ and 3‐year PFS or OS corresponding to a patient's combination of covariates.

**FIGURE 7:**
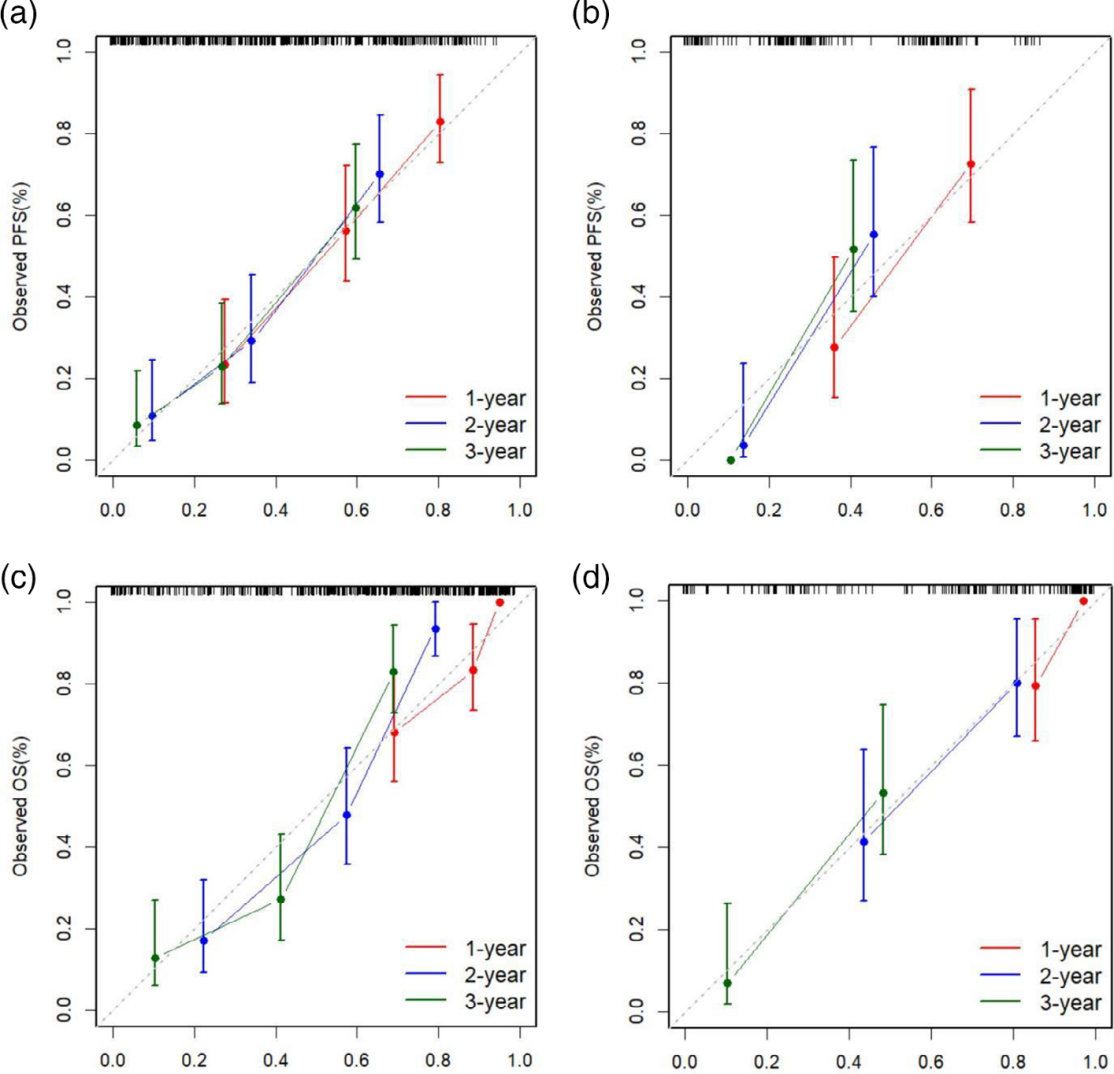
The calibration curve of the nomogram for prediction of 1‐, 2‐ or 3‐year progression‐free survival in the training (a) and validation groups (b). The calibration curve of the nomogram for prediction of 1‐, 2‐ or 3‐year overall survival in the training (c) and validation groups (d). The diagonal line represents an ideal evaluation, while the red, blue or green lines represent the performance of the nomogram. Closer fit to the diagonal dotted line indicates a better evaluation.

Based on the multivariate analysis results, five models, namely, the clinical, preradiomics, delta‐radiomics, preradiomics combined, and delta‐radiomics combined models, were established. The ROC curves for each model are shown in Figure 4. The delta‐radiomics combined model achieved good performance in predicting PFS and OS, with AUC values of 0.875 and 0.835 for the validation group, respectively. The C‐index values for the validation group were 0.704 (95% CI: 0.631–0.777) and 0.770 (95% CI: 0.705–0.803) (Table 4). Although the AUC of the preradiomics combined model was 0.8437 in the training group, it was 0.7968 in the validation group and was less stable than the delta‐radiomics combined model. In the training group, the C‐index of the two models was very similar, but in the validation group, two models showed obvious differences, which was 0.743 for the preradiomics combined model and 0.770 for the delta‐radiomics combined model. According to the above finding, we concluded that the preradiomics combined model might be more valuable than the preradiomics combined model. TNM stage, treatment regimen and delta‐Rad‐score were independent predictors of PFS in the delta‐radiomics combined model. Tumor location, TNM stage, treatment regimen and delta‐Rad‐score were independent predictors of OS in the delta‐radiomics combined model (Table 2). The independent predictors of PFS and OS in the preradiomics combined model are shown in Table 2. The delta‐Rad‐score's optimal cutoff value was determined using the ROC curve (PFS: –0.055, OS: −0.078). KM analysis suggested that patients with higher delta‐Rad‐score had poorer PFS and OS than those with lower delta‐Rad‐score (Figure 5). Nomogram plots for predicting PFS and OS were established (Figure 6). The 1‐, 2‐, and 3‐year PFS and OS for each patient could be predicted based on clinically independent prognostic factors and the delta‐Rad‐score. Finally, we generated calibration curves to assess the accuracy of the nomogram model in predicting 1‐, 2‐, and 3‐year PFS and OS in the training and validation groups (Figure 7).

**TABLE 4:** The C‐index (95% CI) of five models. Whether predicting PFS or OS, the delta‐radiomics combined model was the optimal model.

Models	Training cohort (95% CI)	Validation cohort (95% CI)
PFS		
Clinical model	0.705 (0.654–0.756)	0.667 (0.591–0.743)
Preradiomics model	0.651 (0.588–0.714)	0.598 (0.495–0.699)
Delta‐radiomics model	0.667 (0.612–0.722)	0.614 (0.538–0.690)
Preradiomics combined model	0.728 (0.673–0.783)	0.698 (0.618–0.778)
Delta‐radiomics combined model	0.733 (0.684–0.782)	0.704 (0.631–0.777)
OS		
Clinical model	0.753 (0.706–0.780)	0.720 (0.649–0.791)
Preradiomics model	0.655 (0.599–0.711)	0.593 (0.483–0.703)
Delta‐radiomics model	0.633 (0.563–0.703)	0.671 (0.597–0.745)
Preradiomics combined model	0.757 (0.708–0.806)	0.743 (0.663–0.823)
Delta‐radiomics combined model	0.756 (0.709–0.803)	0.770 (0.705–0.803)

Abbreviations: CI, confidence interval; OS, overall survival; PFS, progression‐free survival.

### Correlation of spleen delta‐radiomics with clinical parameters

**FIGURE 8:**
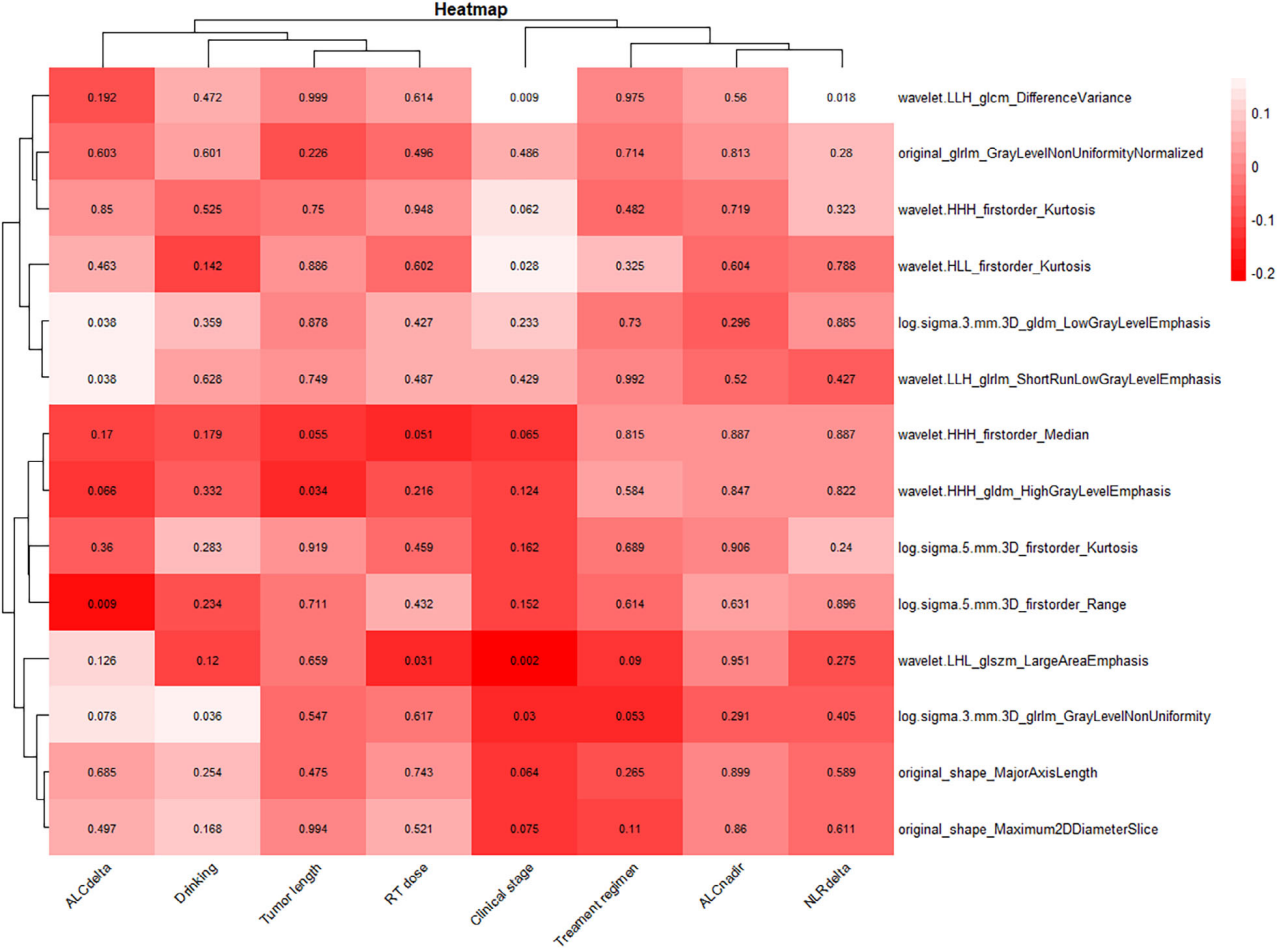
Heat map of associations between selected splenic delta‐radiomic features and clinical factors by using Spearman's correlation analysis. *p* < 0.05 indicates statistical associations as determined using *t*‐tests.

After constructing the prediction model, we explored the correlation of spleen delta‐radiomics with clinical parameters via Spearman correlation analysis and presented it in the form of a heatmap (Figure 8). It revealed that certain spleen delta‐radiomic features were significantly correlated with systemic immune parameters. For example, log.sigma.3.mm.3D_gldm_LowGrayLevelEmphasis, wavelet.LLH_glrlm_ShortRunLowGrayLevelEmphasis, and other features were significantly correlated with delta‐ALC and delta‐NLR.

## DISCUSSION

The spleen plays an important role in systemic antitumor immune response, but whether spleen imaging features have predictive effect for prognosis and immune status was unknown. This study developed splenic CT image‐based survival prediction models for patients with ESCC who had undergone dRT. Several previous studies have demonstrated the utility of radiomics in predicting the survival prognosis of patients with EC.^9^, ^10^, ^11^, ^12^, ^24^, ^25^, ^26^ Delta‐radiomics encompasses a vast amount of time‐dependent information, allowing the dynamic assessment of complete image changes throughout the treatment period. Therefore, we calculated the delta‐radiomics of the spleen to assess its changes before and after radiotherapy. Radiomics modeling based on magnetic resonance images of primary esophageal lesions to predict DFS and OS was established by Chu et al.^24^ The C‐index values of the training and validation groups were 0.714 and 0.729, respectively, and the OS prediction model scores for the respective groups were 0.730 and 0.712. Cui et al.^25^ predicted the PFS and OS of patients undergoing radiotherapy based on esophageal CT images, and the C‐index values were 0.81 and 0.79 for the PFS model and 0.72 and 0.71 for the OS model. Nevertheless, all these studies established their models based on images of the primary esophageal lesion, which more often reflect the biological characteristics of the primary tumor itself. For example, in addition to predicting survival, a previously developed model could also predict early lymph node metastasis.^26^ The radiomic features of the primary tumor may reflect more biological features of the tumor itself, but to understand the systemic immune status before and after treatment, more biomarkers need to be explored, and spleen radiomics is a potential biomarker. Furthermore, our prediction model included independent prognostic factors such as TNM stage, treatment regimen, tumor location and Rad‐score of spleen. TNM stage is an independent predictor which largely represents the situation of the primary tumor which make the prediction effect better. Besides, the model included treatment factors and spleen radiomics which reflect systemic immune response.

Indeed, cancer is a type of systemic disease. Therefore, in addition to primary tumor regression, the systemic immune status is also important for patient outcomes, especially in patients receiving definitive treatment.^16^ Primary tumor radiomics reflect the tumor biological features but do not explain the systemic immune status.^27^, ^28^ Numerous studies have demonstrated the critical impact of systemic immune responses that drive tumor rejection.^16^ The spleen is one of the largest SLOs and major extramedullary hematopoietic organs in the body and may function to modulate the immune system in patients with tumor.^5^ Ugel et al. found that a tumor is able to induce CCR2^+^ inflammatory monocytes, which are characterized by myeloid progenitor cells, to expand in the marginal zone of the spleen, and that these cells cross‐present tumor antigen memory CD 8^+^ T cells to trigger immune tolerance.^29^ Early hematopoietic stem/progenitor cells in the spleen of a homozygous host are functionally altered and thus committed to the production of immunosuppressive myeloid cells.^6^ Zeng et al. found baseline splenic volume to be correlated with peripheral immune cells (lymphocytes, NLR, CD4^+^ T cells, and natural killer cells) in patients with gastric cancer.^30^ In addition, the spleen of patients with tumor develops differently after receiving diverse treatments. Recently, numerous studies have revealed that radiotherapy can cause changes in both the immune environment and splenic volume.^29^, ^31^, ^32^ Wen et al. found that patients with locally advanced non‐small cell lung cancer who underwent radiotherapy combined with platinum‐based chemotherapy exhibited a significant reduction in splenic volume. Injection of low‐dose chemotherapeutic agents, such as gemcitabine and 5‐fluorouracil, in a hormonal host prevented the accumulation of myeloid‐derived suppressor cells by restoring CD 8^+^ T cells in the splenic niche.^29^ Overman et al.^31^ found that 86% of patients with colorectal adenocarcinoma treated with adjuvant FOLFOX had an increase in splenic volume, with 24% experiencing an increase >50%. Kapoor et al.^33^ detected significant reductions in CD 3^+^, CD 4^+^, and CD 8^+^ T cells in the spleen of mice after head or thorax irradiation, accompanied by a decrease in splenic weight and size. In the era of immunotherapy, several studies have proposed that the spleen can serve as a barometer of the systemic immune response during immunotherapy.^34^, ^35^ Knudson et al.^35^ observed an increase in the percentage of CD 8^+^ T and natural killer cells in the spleen of mice treated with anti‐programmed death‐ligand 1. A similar phenomenon was observed in a study by Ji et al.^34^ wherein splenic changes were found to occur during tumor development and after various treatments, thus stimulating our interest in the spleen radiomic of patients with ESCC. Indeed, understanding these systemic immune consequences is important for designing strategies that augment rather than impede anti‐tumor immune responses.

Research in spleen radiomics is limited. Li et al.^13^ investigated the value of splenic radiomic features in predicting recurrence after radical resection of hepatocellular carcinoma and found that a hybrid model of microvascular invasion, tumor radiomic features, and splenic radiomic features for predicting early recurrence achieved the highest C‐index values of 0.780 (95% CI: 0.728–0.831) and 0.776 (95% CI: 0.716–0.836) for the training and validation groups, respectively. On analyzing late recurrence, splenic radiomic features were the only prognostic factors associated with the recurrence of advanced hepatocellular carcinoma. Wang et al.^14^ found splenic radiomic features (*p* = 0.042), age (*p* < 0.001), tumor location (*p* = 0.002), and pTNM stage (*p* < 0.001) to be independent risk factors for survival. Their prognostic prediction model incorporating splenic radiomic features significantly improved the accuracy of the prognostic prediction of 1‐ and 3‐year OS. However, these studies did not explore the relationship between spleen radiomics and the immune system any further, and whether spleen radiomics can truly reflect the body's immune status remains unknown. Therefore, we acknowledged the emerging technology of radiomics and proposed that spleen radiomics could be a promising noninvasive biomarker for predicting the survival and immune status of patients with ESCC.

Radiation‐induced lymphopenia (RIL) is a concern and has been shown to correlate with poor prognosis.^36^ In patients with EC, the incidence of grade 4 RIL was found to reach 11.1%–33.3%.^37^ Several studies have demonstrated an independent association between grade 4 RIL and poorer prognosis in patients with EC.^38^, ^39^ This phenomenon suggests that an organism's systemic immunity influences patient prognosis, prompting us to further explore the correlation between spleen radiomics and peripheral blood immune parameters. The results revealed that splenic delta‐radiomic features were significantly correlated with delta‐ALC and delta‐NLR, indicating that the changes in spleen radiomics in patients with tumor may reflect systemic immune status.

To the best of our knowledge, this is the first study to predict the survival of patients with ESCC via spleen radiomics. We constructed a model and elucidated the possible mechanisms. Moreover, we found that changes in spleen radiomics reflect changes in the systemic immune status, which is worthy of further exploration for the prediction of efficacy and prognosis, among other aspects. Further studies are required to augment current knowledge in this field by investigating the different components of the antitumor immune response and their dynamicity during treatment.

In addition, the study had certain limitations, such as approximately 15% of patients in our study received dRT only. The change of spleen radiomics provides more information associated systemic immune reaction due to different treatment since every antitumor regimen have its double effect, both direct killing tumor and indirect activating immune action. RT could induce more cancer vaccine effect and inducing significant immune reaction than chemotherapy, especially for ESCC, which was cold tumor for immunotherapy. Therefore, this model has its value and could be further studied in the patients underwent routine standard care of dCRT. Furthermore, immunotherapy may be used in the future for the treatment of unresectable ESCC and we should pay more attention to the relationship between splenic imaging and immunotherapy. Since the antitumor mechanism of immunotherapy depends on the participation of immune cells, which would be significantly influenced by spleen. As a result, we believe spleen radiomics might play more important role in the future prognosis prediction for ESCC and further studies are needed.

## AUTHOR CONTRIBUTIONS

Minghuan Li and Ao Liu: Conceptualization. Longxiang Guo; Methodology. Longxiang Guo, Xiaotao Geng and Zongxing Zhao: Software. Longxiang Guo, Yu Nie and Lu Wang: Formal analysis. Defeng Liu, Yuanlin Li and Yi Li: Data curation. Longxiang Guo, Dianxing Li, Qiankun Wang, Zhichao Li and Xiuli Liu: Writing original draft preparation. Minghuan Li and Ao Liu: Writing review and editing. Minghuan Li: Funding acquisition. Longxiang Guo and Ao Liu contributed equally to this study. All authors have read and agreed to the published version of the manuscript. All authors read and approved the final manuscript.

## FUNDING INFORMATION

This study was supported by the National Natural Science Foundation of China (grants 82172677).

## CONFLICT OF INTEREST STATEMENT

The authors declare no conflict of interest.

## Data Availability

The datasets used and/or analyzed during the current study are available from the corresponding author on reasonable request.
